# A precision medicine approach to primary immunodeficiency disease: Ataluren strikes nonsense mutations once again

**DOI:** 10.1016/j.ymthe.2025.03.045

**Published:** 2025-03-28

**Authors:** Laura Lentini, Riccardo Perriera, Federica Corrao, Raffaella Melfi, Marco Tutone, Pietro S. Carollo, Ignazio Fiduccia, Andrea Pace, Davide Ricci, Francesco Genovese, Alain Colige, Philippe Delvenne, Bodo Grimbacher, Michel Moutschen, Ivana Pibiri

**Affiliations:** 1 Department of Biological, Chemical and Pharmaceutical Sciences and Technologies (STEBICEF), University of Palermo, Palermo, Italy; 2 Department of Diagnostic Laboratory, U.O.C. of Pathological Anatomy “G. F. Ingrassia” Hospital, ASP Palermo, Palermo, Italy; 3 Laboratory of Connective Tissues Biology, GIGA Institute, University of Liège, Liège, Belgium; 4 Fonds National de la Recherche Scientifique, Liége, Belgium; 5 Department of Pathology, Centre Hospitalier Universitaire of Liège, Liège, Belgium; 6 Laboratory of Experimental Pathology, GIGA Institute, University of Liège, Liège, Belgium; 7 Institute for Immunodeficiency, Center for Chronic Immunodeficiency (CCI), Medical Center, Faculty of Medicine, Albert-Ludwigs-University of Freiburg, Freiburg, Germany; 8 Clinic of Rheumatology and Clinical Immunology, Center for Chronic Immunodeficiency (CCI), Medical Center, Faculty of Medicine, Albert-Ludwigs-University of Freiburg, Freiburg, Germany; 9 DZIF – German Center for Infection Research, Satellite Center Freiburg, Freiburg, Germany; 10 CIBSS – Centre for Integrative Biological Signalling Studies, Albert-Ludwigs University, Freiburg, Germany; 11 RESIST – Cluster of Excellence 2155 to Hanover Medical School, Satellite Center Freiburg, Freiburg, Germany; 12 General Internal Medicine and Clinical Immunology, Centre Hospitalier Universitaire of Liège, Liège, Belgium; 13 Immunobiology, GIGA Institute, University of Liège, Liège, Belgium

**Keywords:** premature termination codon, nonsense mutation, genetic disorder, LRBA gene, translational readthrough-inducing drugs, oxadiazoles, precision medicine, ataluren, primary immunodeficiency

## Abstract

Primary immunodeficiency diseases (PIDs) are associated with multiple genetic alterations including mutations of the lipopolysaccharide responsive Beige anchor (LRBA) gene. Nonsense mutations in the LRBA gene resulting in premature termination codons cause the loss of LRBA protein expression in PID. We evaluated the impact of a translational readthrough-inducing drug (TRID) ataluren as a nonsense suppression therapy in a PID patient with a homozygous stop codon mutation in exon 30 of LRBA. A precision medicine approach allowed us to pass from “*in silico*” to “*in vitro*” to the “bedside”: following the *in vitro* treatment of patient-derived primary fibroblasts with ataluren, we observed a restoration of the LRBA protein expression and localization. *In silico* predictions suggested LRBA retained function after readthrough. Based on the successful experimental and computational results we treated the patient with ataluren resulting in an improvement of his clinical symptoms and quality of life. Importantly, the clinical symptoms were associated with a recovery of LRBA expression in liver biopsies post-treatment compared with pre-treatment. Our results provide a proof of concept demonstrating that ataluren, can rescue LRBA expression in PID. This work highlights the potential for personalized precision medicine approaches to be exploited for different genetic diseases due to premature termination codons.

## Graphical abstract

**Figure:**
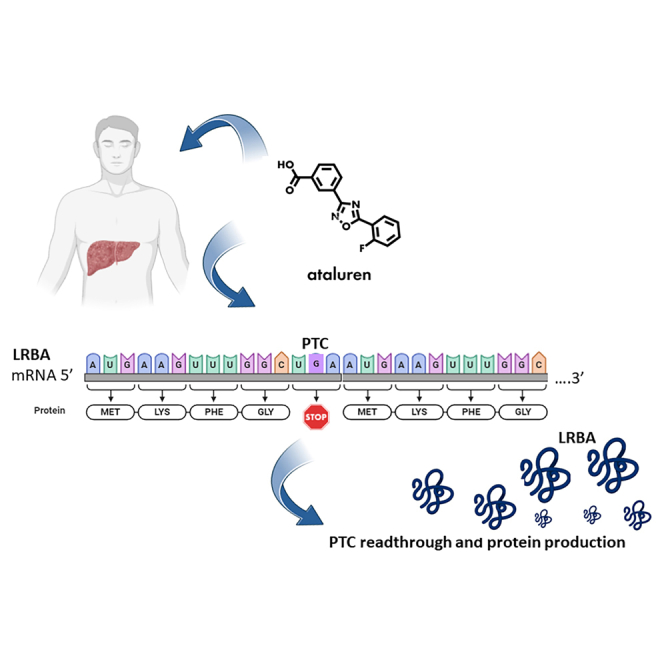

## Teaser

Ataluren, a translational readthrough-inducing drug (TRID), was evaluated in a PID patient with a homozygous stop codon mutation in the LRBA gene. *In vitro* treatment restored LRBA protein expression and function, and clinical improvements were observed post-treatment, highlighting the potential of precision medicine for genetic diseases with premature termination codons.

## Introduction

Primary immunodeficiency diseases (PIDs) are rare genetic diseases characterized by susceptibility to infections, increased risk of autoimmunity, hypogammaglobulinemia, lymphoproliferative syndromes, chronic diarrhea, and functional alteration in T cells.^1^^,^^2^ About 400 known gene alterations are known to be associated with PIDs, among which are mutations of the LRBA gene, encoding a widely expressed multi-domain protein with highly conserved BEACH (Beige and Chediak-Higashi) domain, a region implicated in membrane trafficking, that is present in a family of proteins conserved throughout eukaryotes. This is involved in the regulation of endosomal trafficking, particularly endocytosis of ligand-activated receptors. LRBA deficiency accounts for a fair fraction of monogenic antibody deficiency.^3^^,^^4^^,^^5^^,^^6^ Among the pathogenic variants of LRBA, more than 20% correspond to single nucleotide nonsense mutations.

The LRBA gene maps to chromosome 4 (4q31.3) and is 750 kb. The gene is transcribed into an mRNA of 61 exons, encoding a 2,863-amino acid protein with a mass of 319 kDa.^3^^,^^4^ LRBA protein is a cytosolic protein that localizes in the endoplasmic reticulum, trans-Golgi apparatus, endocytosis vesicles, and lysosomes, and it is ubiquitously expressed by almost all cell types. However, it is highly expressed in immune cells.^1^

Several germline mutations have been identified in the *LRBA g*ene in patients suffering from various clinical symptoms. In particular, the nonsense mutations identified result in the lack of LRBA protein expression and are associated with a spectrum of phenotypes such as autoimmunity, chronic diarrhea, B cell deficiency, hypogammaglobulinemia, functional alteration in T cells, and aberrant autophagy.^2^^,^^5^

In disorders associated with nonsense mutations, a promising therapeutic approach to restore the expression of a premature termination codon (PTC)-harboring mRNA is the use of Translational readthrough-inducing drugs (TRIDs).^7^^,^^8^^,^^9^^,^^10^ The activity of TRIDs relies on the misreading of the premature termination signal by inserting a near-cognate amino acid in the place of the PTC, so allowing the translation to continue and produce a full-length polypeptide.^11^ In the last 10 years, several molecules have been identified with this aim. Aminoglycoside antibiotics have been the first molecules found to have a readthrough effect on premature termination codons (PTCs). Ataluren was the first non-aminoglycoside small molecule to show this specific activity. In contrast, ataluren has no antibiotic activity but is able to promote the readthrough of PTCs, particularly UGA,^12^^,^^13^^,^^14^ thus being a good candidate for developing a therapy for nonsense mutation diseases.

Ataluren does not affect nonsense-mediated decay (NMD) directly but may benefit from a synergistic effect when combined with NMD inhibitors.^15^ Its mechanism of action has been recently elucidated and is interestingly peculiar, as it inhibits the termination activity of the release factor complex (RFC) while preserving the productive binding of the near-cognate ternary complex (TC; aa-tRNA•eEF1A•GTP).^16^

Phase I and II clinical trials, including healthy subjects and nonsense mutation mediated Cystic Fibrosis (nmCF) and nonsense mutation mediated Duchenne muscular dystrophy (nmDMD) patients, have demonstrated the long-term safety of ataluren. Following an open-label Phase IIa study of ataluren (ClinicalTrials.gov identifier: NCT00264888) that demonstrated increases in full-length dystrophin expression in patients with nmDMD treated with ataluren for 28 days, ataluren was evaluated in patients with nmDMD in Phase IIb (ClinicalTrials.gov identifier: NTC00592553) and Phase III (ClinicalTrials.gov identifier: NCT01826487) randomized, double-blind, placebo-controlled trials.^17^ These trials showed a benefit of ataluren (40 mg/kg per day) compared with a placebo in a range of clinical endpoints.^17^

Beyond nmDMD and nmCF, there is limited clinical evidence supporting ataluren’s efficacy in other diseases caused by nonsense mutations. Diverse studies suggest potential applicability across various genetic disorders with premature stop codons^18^^,^^19^^,^^20^; however, further research and clinical trials are necessary to establish ataluren’s effectiveness in other conditions.

The drug was conditionally approved by the European Medicines Agency (EMA) for use in nmDMD patients.^21^ Nevertheless, due to clinical factors in the nmDMD patient population, the benefits of ataluren remain a topic of debate. In fact, in a recent communication (October 2024), the EMA reaffirmed its recommendation not to renew its conditional marketing authorization in nmDMD patients, citing a lack of confirmed effectiveness. However, numerous patients have reported positive outcomes with this therapy, and international patient advocacy groups continue to contest this decision. In the meantime, the US Food and Drug Administration (FDA) has accepted the resubmission of the New Drug Application (NDA) for Translarna (ataluren) for the treatment of nmDMD. The FDA’s evaluation process for the approval request has begun, which could potentially lead to the availability of the treatment for patients in the United States if a favorable decision is reached. This study aims to contribute to the ongoing discussion by providing insights that may clarify ataluren’s potential clinical benefits and inform future evaluations.

Herein, we report the evaluation of the molecular and clinical impact of ataluren in a 39-year-old PID patient harboring a homozygous stop mutation (c. 5047) (C>T) in the LRBA gene.

The patient had a complex phenotype including autoimmune bicytopenia, granulomatous lymphocytic interstitial lung disease, non-cirrhotic portal hypertension, and severe diarrhea. Due to the patient’s homozygous premature stop codon mutation, ataluren was considered as a potential therapy. The *in vitro* treatment of patient primary fibroblasts with ataluren demonstrated the rescue of the LRBA protein expression and its correct localization. Moreover, *in silico* predictions by virtual mutagenesis of the LRBA protein structure encouraged the hypothesis of preservation of protein functionality after readthrough.

Based on the successful *in vitro* and *in silico* results, we obtained the authorization to perform a single trial for off-label use and we started the patient treatment with ataluren. The treatment resulted in an improvement of the patient clinical symptoms and re-expression of LRBA protein in the patient’s liver highlighting the therapeutic potential of ataluren in this clinical case.

## Results

### LRBA protein translation is rescued following ataluren treatment

**Figure 1:**
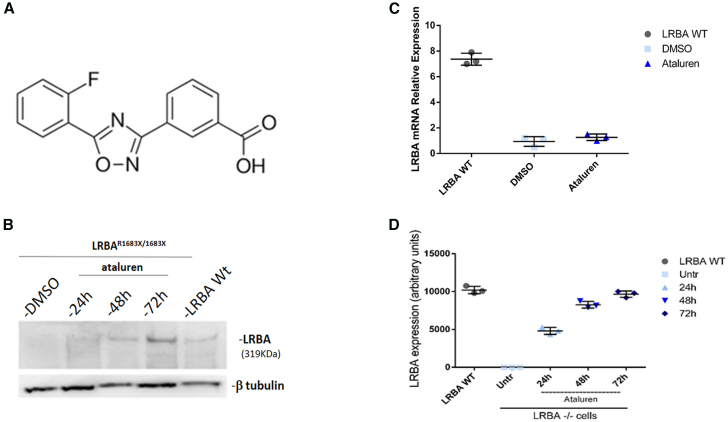
Ataluren structure and *in vitro* analysis to assess its activity in nonsense LRBA human primary fibroblasts (A) Ataluren structure. (B) LRBA gene expression in patient-derived LRBA^R1683X/R1683X^ primary fibroblasts by quantitative real-time RT-PCR analysis. The graph shows LRBA expression in the positive control (IMR-90 cell line), negative control (DMSO-treated patient’s cells), and ataluren (12 μM) treated samples. A significant variation was highlighted in the positive control. *p* value (*p* < 0,0001) was calculated by the one-way ANOVA test. (C and D) Western blot to detect LRBA protein in LRBA^R1683X/R1683X^ primary fibroblasts untreated or treated with ataluren 12 μM for 24, 48, and 72 h. The positive control (LRBA WT) is represented by protein extract in embryonal fibroblast cells (IMR-90). Beta-tubulin was used to control the loading. ImageJ software performed LRBA band quantification.

To evaluate the ability of ataluren to rescue LRBA expression, we used LRBA^R1683X/R1683X^ fibroblasts from a biopsy of the patient affected by PID. Cells were treated for 24, 48, and 72 h with 12 μM ataluren (Figure 1A) and total RNA and proteins were extracted and analyzed by real-time RT-PCR and western blot analysis (Figures 1B–1D).

We decided to perform a real-time RT-PCR on RNA extracted after 72 h of chronic treatment to investigate if there was a change in the mRNA expression levels and its stabilization. As shown in Figure 1, the ataluren-treated sample was compared with the vehicle-treated cells and to the wild type; precisely, IMR-90 cell lines have been chosen as wild-type positive control.

Real-time RT-PCR did not show an increase in mRNA levels in LRBA^R1683X/R1683X^-treated cells when compared with the control (vehicle). Moreover, it showed a significant difference in LRBA mRNA expression between wild-type and LRBA-mutated cells (Figure 1C).

As shown in Figures1B and 1D, ataluren rescued LRBA protein expression by increasing its levels starting at 24 h of treatment when compared with the untreated cells. Strikingly, the increase was time-dependent, as observed at 48 and 72 h of treatment.

**Figure 2:**
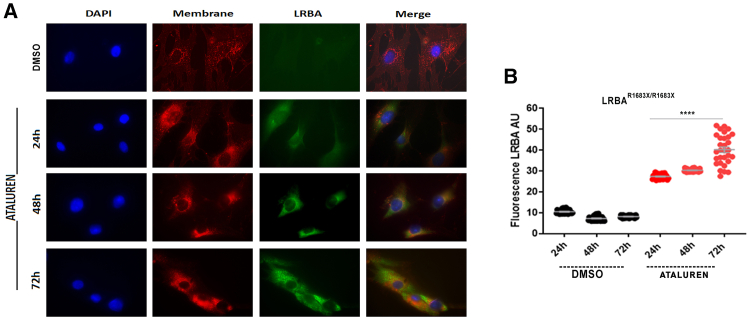
Analysis of the expression and localization for the LRBA rescued protein after treatment with ataluren (A) Immunofluorescence analysis of LRBA^R1683X/R1683X^ primary fibroblasts. Cells were treated with ataluren for 24, 48, and 72 h at 12 μM. LRBA protein (green) was revealed by a specific primary antibody and secondary antibody (Alexa 488). Nuclei (blue) were stained by DAPI (4′,6-diamidino-2-phenylindole), and plasma membranes (red) were stained by wheat germ agglutinin (WGA) conjugated to Alexa 594. (B) Quantification of the fluorescence of the LRBA^R1683X/R1683X^ primary fibroblasts. *p* value (*p* < 0.0001) was calculated by one-way ANOVA test.

**Figure 3:**
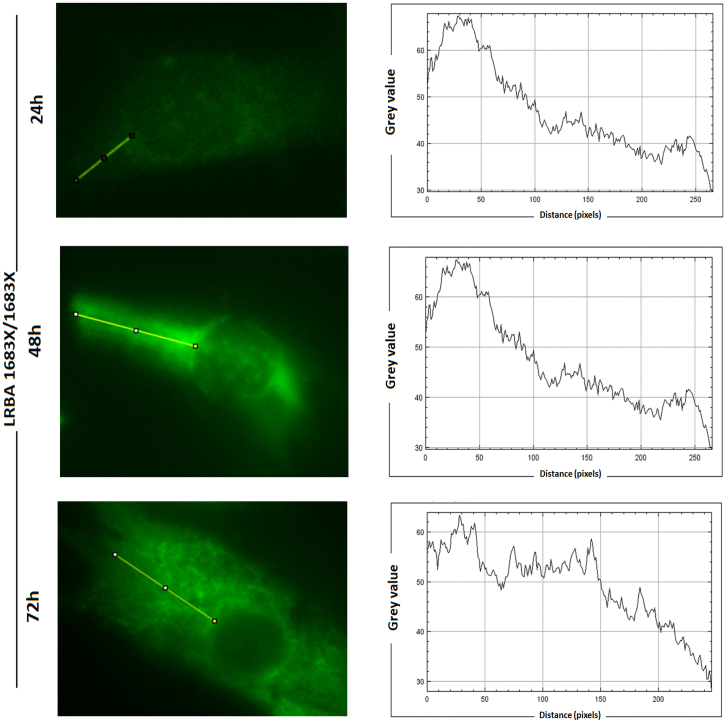
Measurement of the fluorescence diffusion in the cytoplasm of LRBA^R1683X/R1683X^ primary fibroblasts after the indicated treatments

Moreover, LRBA expression and localization were also evaluated by immunofluorescence analysis. The increased LRBA protein levels were paralleled by its increased subcellular localization. Indeed, LRBA is initially localized at the level of the endoplasmic reticulum and Golgi apparatus (48 h ataluren, Figures 2 and 3) to finally reach a more complete cytoplasmic localization at 72 h of ataluren treatment (Figures 2A and 3).

Taken together, our results suggest the effectiveness of ataluren to exert its readthrough activity on the translation process to rescue high levels of LRBA protein in LRBA^R1683X/R1683X^.

### Prediction of the LRBA protein structure

Considering that ataluren readthrough could promote the insertion of a near-cognate tRNA/amino acid (namely, tryptophan in the majority of the cases) in the PTC misreading,^10^^,^^22^^,^^23^ we performed a virtual analysis of the LRBA protein structure by replacing arginine with tryptophan in the readthrough of the PTC. This simulation allowed us to hypothesize that LRBA protein activity, after readthrough by ataluren, should not be affected.

The complete experimental full structure of lipopolysaccharide-responsive and beige-like anchor protein (LRBA) has not yet been resolved. Starting from the primary structure of LRBA containing 2,863 amino acids obtained from the UniprotKB in FASTA format (P50851), comparative modeling using the SWISS model was performed first. The best homology model was obtained by the PDB ID template 1T77 showing a sequence identity of 99.6% but limited to the domain 2076-2489. It is known that the nonsense mutation responsible for LRBA activity loss is R1683X, which falls out of the modeled region using comparative modeling. Given this limitation, we decided to perform an *ab initio* modeling of the domain within the region surrounding the nonsense mutation. One of the problems of the *ab initio* modeling of the protein was related to decreasing accuracy of the prediction with the sequence used in the model, so two sequences of 100 residues, from position 1633 to position 1733 with respect to the R1683X, were constructed. It is known that a PTC UGA was bypassed with the insertion of Trp (W), Arg (R), or Cys (C); however, experimental evidence showed that the preferred insertion is Trp.^22^

**Figure 4:**
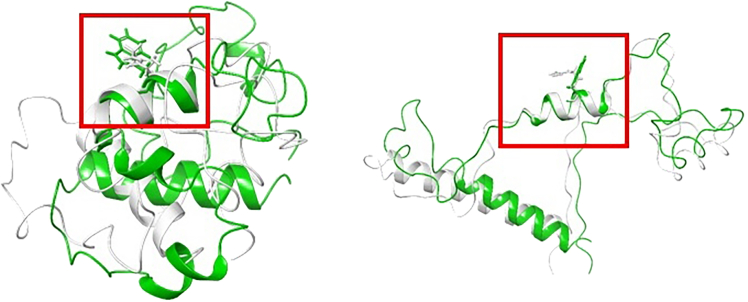
Models obtained by C-QUARK, the native sequence with R1683 residue are reported in gray ribbons and sticks, respectively The near-cognate sequence, with W1683 residue, is reported in green ribbons and sticks, respectively (left); models obtained by Robetta, the natural sequence with the R1683 residue, are reported in gray ribbons and sticks, respectively. The near-cognate sequence with W1683 residue is reported in green ribbons and sticks, respectively (right). The red line squares focus on the α-helix where the R1683W falls.

The first sequence bears the natural amino acid R and the second one bears the near-cognate amino acid W. These sequences have been modeled by using two protein prediction structure tools: Robetta and C-QUARK. The C-score (Confidence score) and the TM-score (Template-modeling score) calculated by C-QUARK are −1.093 and 0.402 ± 0.144, respectively, for the native sequence. The predicted secondary structure showed a high confidence score, 9 out of 10, in the +3 and −5 sequence from the R1683 residue. This high confidence value is due to the prediction of an α-helix as a highly structured region of the protein. The C-score and the TM-score calculated by C-QUARK are −1.311 and 0.393 ± 0.147, respectively, for the near-cognate sequence. Also in this model, the predicted secondary structure showed a high confidence score, 9 out of 10, in the +3 and −5 sequence from the R1683W. The comparison of the two models obtained by C-QUARK showed that the key residue falls in a predicted α-helix and the substitution with a W residue seems to restore the structure of the native protein (Figure 4 left). The predicted structures obtained by Robetta of the natural sequence showed a confidence score of 0.56/1 and 0.57/1. These results were corroborated by low RMSD values (2.99 Å for C-QUARK, 1.71 Å for Robetta) for the substructure −4 to +4 residues from the 1683 position. Even though the predicted models by C-QUARK and Robetta show a quite different tertiary structure, it is worth noting that in both models the R1683W region was predicted to assume an α-helix structure (Figure 4 right).

### Ataluren treatment induces an improvement of the PID patient’s symptoms

The 39-year-old male harboring a homozygous nonsense mutation (c. 5047C>T; p.Arg1683∗) in the *LRBA* gene has had numerous complications during his life, including molluscum contagiosum in the perineal area and respiratory infections since the age of 12. At the age of 16, he was hospitalized for idiopathic thrombocytopenic purpura, autoimmune anemia, and severe diarrhea. On that occasion, atrophic gastritis was also found, with antibodies directed against intrinsic factor. Clinical analysis evidenced the absence of immunoglobulin (Ig)A and lowered IgG levels to 3.5 g/L. Substitution with immunoglobulins was initiated, but numerous complications continued to occur, including several episodes of granulomatous lymphocytic interstitial lung disease (GLILD), an inflammatory lesion of the central nervous system in 2008, and nodular regenerative hyperplasia of the liver with non-cirrhotic portal hypertension in 2014. A portosystemic shunt had been placed for recurrent ascites but had to be removed due to respiratory deterioration. Moreover, portal hypertension had worsened in 2019 due to a partial thrombosis of the portal vein.

However, it is chronic and severe diarrhea that has been the major symptom of this patient since the age of 16, with stool frequency up to 10 per day and a 24-h stool weight ranging between 1,000 and 5,000 g/24 h. Alpha-1-antitrypsin clearance was normal, thus excluding exudative enteropathy. All biopsies revealed an aspect of nonspecific colitis. Numerous treatments have been tried (methylprednisolone, budesonide, infliximab, vedolizumab, rapamycin, ustekinumab, and abatacept). Abatacept had brought some respiratory improvement, but severe diarrhea persisted at the initiation of ataluren treatment.

Immunological exploration showed typical abnormalities of common variable immunodeficiency, such as low values of switched memory B cells and increased values of transitional B cells. There was also a reduced concentration of regulatory T cells. Interestingly, membrane expression of CTLA4 on T cells was comparable to that of control subjects. Finally, there was a very increased value of sCD25 (a marker often considered as related to disease activity).

Given the nonsense homozygous mutation, ataluren emerged as a prospective therapeutic option.

*In vitro* tests with the patient-derived fibroblasts treated with ataluren showed expression of LRBA protein, prompting us to evaluate the potential use of ataluren administration to this patient. Following the approval of treatment, ataluren was started in July 2021.

Ataluren was given at a dose of 500/500/1,000 mg. When this treatment was introduced, the patient was already being treated with abatacept 125 mg per week and methylprednisolone 16 mg every day. Tolerance proved to be excellent following 2 years of treatment. Very interestingly, the patient’s diarrhea resolved rapidly with a reduction in the number of stools 2 weeks after starting treatment and normalization of complaints 2 months after the start of ataluren administration. Also noteworthy was a significant improvement in thrombocytopenia under ataluren. However, pre-existing portal vein thrombosis and nodular regenerative hyperplasia obliged to liver transplant.

**Figure 5:**
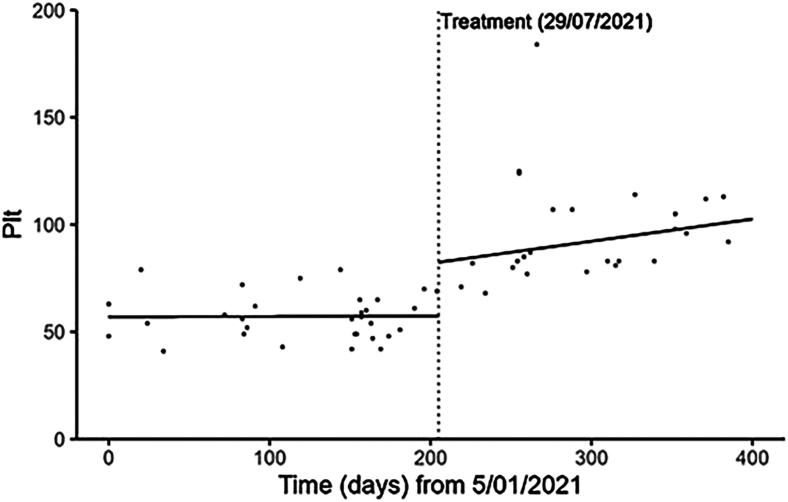
Ataluren improved thrombocytopenia in the patient: Platelet evolution before and after the start of Ataluren treatment

It is worth underlining that liver transplantation, that was formerly denied to the patient due to his severe clinical conditions, was made possible after ataluren treatment and improvement of the patient clinical conditions. The patient was deemed eligible, given the disappearance of diarrhea and the improvement in general condition. The transplant was performed on January 25, 2022. To date, the patient remains on ataluren at the same dose and has not presented any new diarrheal episode or other complications (particularly respiratory) such as GLILD. Biological evaluation has shown a progressive reduction in sCD25 levels (Table 1); moreover, as stated above, platelets significantly increased after treatment (Figure 5); however, the abnormalities of the lymphocyte phenotype remained unchanged (Table 1).

**Table 1:** Ataluren improved general biological parameters in the patient: The most significant biological values at day 0 and week 100 following the initiation of treatment are illustrated

	Units	Normal values	Day 0	Wk 100
B cells (CD19)	% of lymphocytes	5–20	6.00	6
Absolute B cells	/mL	70–520	120.00	80
Transitional (IgM++ IgD++ CD38++)	% of CD19	1.6–3.9	13.70	10.7
Transitional B cells (absolute)	/mL	2–10	16.44	8.56
Switched memory B cells (IgM-IgD-CD27+)	% of CD19	6.6–21.1	0.10	0
Switched memory B cells (absolute)	/mL	8–54	0.12	0
CD21lo B cells (CD21lo CD38lo)	% of CD19	1.7–8.0	4.80	1.2
CD21lo B cells (absolute)	/mL	3–15	5.76	0.96
T cells (CD3)	% of lymphocytes	60–87	82.00	87
T cells (absolute)	/mL	600–2460	705.20	1157
CD4 T cells	% of lymphocytes	32–61	44.00	45
CD4 T cells (absolute)	/mL	490–1670	378.40	630
CD8 T cells	% of lymphocytes	14–43	34.00	38
CD8 T cells (absolute)	/mL	220–1110	292.40	530
CD4/CD8			1.29	1.19
Regulatory T cells	% of CD4	5.3–11.5	na	2.9
Regulatory T cells (absolute)	/mL	38–137	na	18
IgA	g/L	0.63–4.84	0.00	0
IgM	g/L	0.22–2.40	0.23	0.08
Beta2 microglobulin	mg/L	0.97–2.64	5.17	3.78
sCD25	pg/mL	<2000	8505.00	3019

### Increase of LRBA protein expression in the patient’s liver sample after prolonged ataluren treatment

Explant liver biopsy, performed 6 months after starting ataluren treatment, confirmed the re-expression of LRBA protein, demonstrating the therapeutic potential of ataluren in PID due to homozygous nonsense mutation in the LRBA gene.

**Figure 6:**
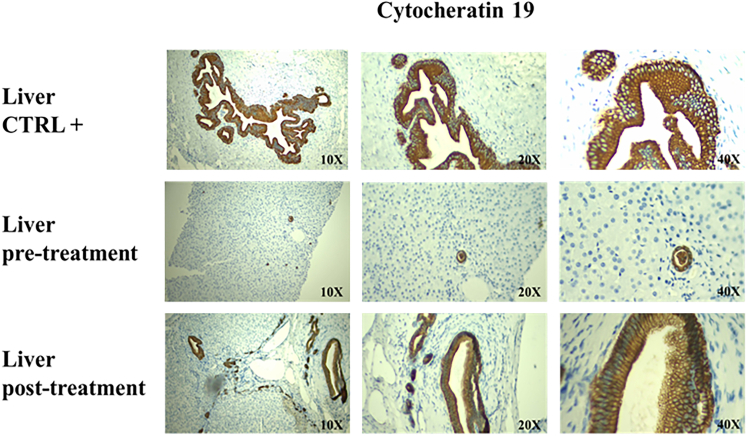
Cytokeratin 19 (clone A53-B/A2.26, Roche Ventana) immunostaining is shown in the indicated samples (positive, healthy control, pre-and post-treatment liver samples) Three magnifications are reported (10×, 20×, 40×). This figure shows that all samples are responsive to the immunostaining and the protocol used, since cholangiocytes of hepatic ducts are stained.

All tissue sections analyzed were immunohistochemically reactive, since antibody anti-cytokeratin 19, used as epithelial control marker, stained the cholangiocytes of hepatic ducts intensively (Figure 6). Importantly the anti-LRBA antibody, as expected, specifically stained the endothelial and glandular cells in hepatocytes and cholangiocytes in tissue sections of healthy donor liver, strongly and weakly, respectively.

**Figure 7:**
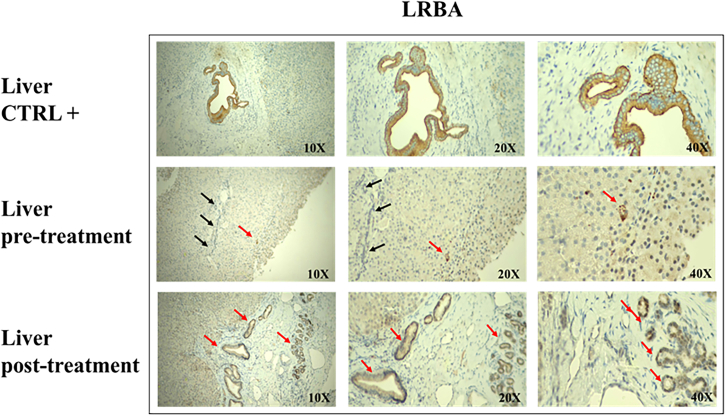
LRBA protein detection in the indicated samples (positive, healthy control, pre- and post-treatment liver samples) The tissues were incubated at 36°C with the antibody anti-LRBA 1:200 for 36 min. At the bottom, magnifications of positive control samples show the level of LRBA expression of a healthy condition liver. In the middle, the detection of LRBA protein is weaker than in the healthy and post-treatment samples, as indicated by the red arrow, contrary to the black arrows, showing the lower intensity of the signal in other hepatic ducts. Last, in the lower part, the samples derived from the post-treatment liver are shown. In red arrows, the major intensive detection of LRBA protein in hepatic ducts. Also, weak staining is distinguishable in all samples.

The anti-LRBA antibody, in both analytical samples, pre-and post-treatment, marked the hepatocytes and cholangiocytes weakly. However, the intensity of the target proteins observed was higher in the post-treatment samples, compared with the pre-treatment (Figure 7).

An immunohistochemical staining in tissue derived from the patient pre-ataluren treatment, was weaker relative to that of the healthy control and the post-ataluren treatment samples. This lower-intensity pre-treatment is most likely explained by the reduced production of LRBA protein in the pathological samples. Moreover, post-treatment samples have shown relatively higher levels of LRBA protein, as indicated by an elevated number of hepatic ducts stained.

## Discussion

This approach can be considered a personalized precision medicine approach, since *in vitro* test of ataluren, on patient-derived *LRBA*^*R1683X/R1683X*^ primary fibroblasts, allowed us to demonstrate that this TRID, currently in use for nonsense mutation-mediated Duchenne muscular dystrophy (nmDMD) patients, can rescue the mature form of LRBA protein (268 kDa) as shown by western blot analyses after chronic treatments, despite the lack of a significant increase in LRBA mRNA levels. Moreover, immunofluorescence analysis showed the correct cytoplasmic localization of the rescued LRBA, supporting that ataluren drives the ribosomal readthrough on the LRBA^*R1683X/R1683X*^ mRNA.

The *in silico* approach allowed the comparison by two models obtained by C-QUARK and Robetta, of the native LRBA and LRBA rescued by readthrough, showing that the key residue falls in a predicted α-helix, and the substitution with a W residue (the most likely introduced by UGA readthrough) seems to restore the secondary structure of the native protein. Even though the predicted models by C-QUARK and Robetta show a quite different tertiary structure, it is interesting that the R1683W region was predicted to assume an α-helix structure as the native sequence.

These encouraging results prompted us to explore the potential activity of ataluren on the patient in a single clinical trial.

The treatment was exceptionally well-tolerated, with a current follow-up of over 2 years. Severe diarrhea, a cardinal symptom that had not responded to other treatments (including abatacept), entirely resolved in the weeks following the initiation of therapy.

Importantly, the clinical symptoms were associated with a recovery of LRBA expression and localization in liver biopsies post-treatment compared with pre-treatment, by immunohistochemical analyses.

This case report allows for several significant observations. First, it marks the inaugural instance of employing a translational readthrough approach in a patient with common variable immunodeficiency, specifically a deficiency in LRBA (one of the most severe forms in this disease family). Second, this is the first account of the re-expression of a gene affected by a nonsense mutation in a deep -organ in a patient treated with such an approach.

Focusing on just one patient, our case report has certain limitations: a liver transplant was performed 6 months after starting the ataluren treatment, undoubtedly contributing to the general improvement of the patient’s condition beyond the initial improvement of clinical symptoms at 6 months. Hence, determining the specific long-term clinical benefit of the ataluren treatment is not possible, albeit we must consider that the liver transplant was feasible due to the general improvement of the patient’s condition reached after ataluren treatment.

Notably, soluble CD25 level dropped by more than 60% after 100 days of ataluren treatment. This is compatible with an effect of ataluren on abnormal T cell activation associated with LRBA deficiency. Accordingly, normalization of T cell counts could be linked to reduced activation-induced cell death, a phenomenon described in other conditions such as HIV infection or autoimmune diseases. In contrast, the abnormalities involving humoral immunity remained unchanged, including the IgA deficiency, the significant reduction in switched memory B lymphocytes, or the increase of transitional B cells. The reasons for this irreversibility are unclear: a loss of function of the mutated protein could be considered, although our bioinformatic models do not support this hypothesis. Alternatively, irreversible phenomena of clonal exhaustion could be considered.

Regardless, the resolution of diarrhea under ataluren, despite the continued presence of adaptive immunity anomalies, may suggest that the re-expression of LRBA in other non-hematopoietic (i.e., epithelial cells) or hematopoietic (i.e., innate lymphoid cells) cell types has broadly improved our patient’s intestinal function.

These results highlight the efficacy of ataluren for nonsense mutation diseases beyond nmDMD and support the expansion of TRIDs as drugs for different genetic diseases with nonsense mutations (causing PTCs). Moreover, these results justify the intensification of research to identify patients with nonsense mutation mediated genetic diseases where ataluren may be used to address the medical need for different patient populations using precision medicine approaches.

## Materials and methods

### Chemistry

All solvents and reagents were obtained from commercial sources. Ataluren has been synthesized as reported^13^ and its characterization was in agreement with what was reported in the literature.^12^ The purity grade assessment has been realized by high-performance liquid chromatography-mass spectrometry (HPLC-MS) and was >99%.

IR spectra were registered with a Shimadzu FTIR-8300 instrument. ^1^H NMR spectra were recorded on a Bruker 300 Avance spectrometer, operating at 300 MHz, using the solvent residual peak as a reference.

Reversed-Phase HPLC/ESI/Q-TOF HRMS experiments were performed using mixtures of water and acetonitrile of HPLC-MS grade as eluents with the addition of 0.1% (v/v) of formic acid. The HPLC system was an Agilent 1260 Infinity. A reversed-phase C18 column (Luna Omega 5 μm Polar C18 150 × 2.1 mm) with a Phenomenex C18 security guard column (4 mm × 3 mm) was used. Mass spectra were obtained on an Agilent 6540 UHD accurate-mass Q-TOF spectrometer equipped with a Dual AJS ESI source working in positive mode.

Flash chromatography was performed by using silica gel (Merck, 0.040–0.063 mm) and mixtures of ethyl acetate and petroleum ether (fraction boiling in the range of 40–60°C) in various ratios.

3-[5-(2-fluorophenyl)-[1,2,4]-oxadiazol-3-yl]-benzoic acid (ataluren).

**IR** 1297cm^−1^**;**

^**1**^**H NMR** (d6-DMSO, 300 MHz) δ 13. 39 (br s, 1H), 8.64 (m, 1H), 8.62–8.23. (m, 2H), 8.18–8.14 (br s, 1H), 7.85–7.71 (m, 2H), 7.60–7.47 (m, 2H).

**HRMS:** (m/z), calcd for C_15_H_10_FN_2_O_3_ (MH^+^), 285.06814; found, 285.06797.

### Cell culture

Fibroblasts, derived from patient biopsy, harboring the nonsense mutation c.5047 C>T 8p. R1683X in the *LRBA* gene, were cultured in T-25 flasks for adherent cells with minimum essential medium (MEM) (Gibco–Life Technologies, Waltham, MA), supplemented with 10% FBS (CORNING, New York, US), 1% L-glutamine (200 mM solution, CORNING, New York, US), 1% antibiotics penicillin and streptomycin (Solution 100x, CORNING, New York, US), 1% non-essential amino acids (100 mM solution, CORNING, New York, US), 1% sodium pyruvate (100-mM solution, Gibco–Life Technologies, Waltham, MA). Cells were maintained in a humidified atmosphere of 5% CO_2_ at 37°C. When treated with ataluren, the medium was replaced with MEM supplemented as described above, but without antibiotics.

The treatment with ataluren was chronic, every 24 h the medium was refreshed and ataluren (12 μM) was added.

### RNA isolation and quantitative real-time RT polymerase chain reaction

Cells were harvested 24, 48, and 72 h after treatment with ataluren. Cells were lysed and total RNA was purified using spin column centrifugation with a PureLink RNA Mini Kit (Life Technologies, Waltham, MA), following being transcribed in cDNA with High Capacity cDNA Reverse Transcription kit (Applied Biosystem, Foster City, CA), under the following conditions: 25°C for 20 min, 37°C for 60 + 60 min, 85°C for 5 min, 4°C for ∞. RNA integrity was simultaneously controlled by 1% gel agarose electrophoresis analysis. After that, 1 μg cDNA was used to carry on a real-time RT-PCR experiment, with specific primers designed using Primer-BLAST software (NCBI). Respectively, primer sequences are reported: Forward 5ʹ – TCCGAGCCCTCAATGTGTTC – 3ʹ; Reverse 5ʹ – CATGGCAGAACCTCTGGGAG – 3ʹ. The experiment was performed with Power SYBR Green PCR Master Mix (Applied Biosystem , Foster City, CA) and the 96-well plate analyzed by 7300 Real-Time PCR System (Applied Biosystem TM, Foster City, CA), with the following setup: 1 stage at 95°C for 10 min, the second stage repeated 40 times at 95°C for 15 s, ending with 1 cycle at 60°C for 1 min.

### Protein extraction and western blot analysis

Proteins were extracted by Ripa Buffer (Thermo Scientific, Waltham, MA) and quantified by Bradford colorimetric assay (Thermo Scientific, Waltham, MA). Thirty micrograms of proteins were separated by 3%–8% SDS-PAGE and transferred to PVDF Transfer Membrane (Thermo Scientific, Waltham, MA) by electroblotting overnight (O.N.) at 4°C. The filter was incubated with a rabbit antibody anti-LRBA (Bethyl Laboratories, Montgomery, TX, 1:1,000) O.N. at 4°C, so with a horseradish peroxidase (HRP)-conjugated anti-rabbit antibody (Abcam ab97051 1:5,000) for 1 h at room temperature. The protein of interest was detected using SuperSignal West Femto Maximum Sensitivity Substrate (Thermo Scientific, Waltham, MA), and image acquisition was made with ChemiDoc XRS System (Bio-Rad, Hercules, CA). Protein bands were compared with those of β-tubulin, revealed after monoclonal anti-β-tubulin antibody (Sigma T4026, 1:5,000) incubation O.N., followed by anti-mouse IgG HRP-conjugated antibody (Invitrogen 31430 Goat anti-mouse IgG H + L, peroxidase conjugated, 1:5,000), to confirm the presence of an equal quantity of protein for each lane. Bands were quantified employing ImageJ software.

### Immunofluorescence microscopy

An amount of 4 × 104 cells were seeded on a glass coverslip and fixed with iced methanol for 30 s. The endomembrane system and plasma membrane were stained with the wheat germ agglutinin (WGA) Alexa 594 (Invitrogen W11262, 1:1,000) for 10 min at room temperature, while nuclei were detected with 4′,6-diamidino-2-phenylindole (DAPI) (Life Technologies, Waltham, MA). LRBA was visualized after O.N. incubation at 4°C with a rabbit polyclonal antibody (Bethyl Laboratories, Montgomery, TX, 1:1,000), followed by anti-rabbit IgG (whole molecule)-FITC antibody incubation (Sigma 036K4820 1:200) 1 h at 37°C. Cells were observed and examined under a Zeiss Axioskop microscope (Oberkochen, Baden-Württemberg, Germany), specifically equipped for fluorescence. Areas/fields images and protein expression were analyzed using ImageJ software.

### Prediction of the protein structure simulations

The FASTA sequence of the human isoform 2 of human LRBA (P50851) was retrieved from the UniProtKB.^24^ The SWISS-MODEL^25^ template library (SMTL version 2021-03-03, PDB release 2021-02-26) was searched with BLAST^26^ and HHblits^27^ for evolutionary-related structures matching the target sequence. The prediction of the protein structure was performed using two different methods: Rosetta, a prediction server developed by the Baker lab at the University of Washington. Rosetta’s primary service is to predict the 3-dimensional structure of a protein given the amino acid sequence. The option used for structure prediction was the deep learning-based method, RoseTTAFold^28^; C-QUARK. The pipeline is established on the framework of the QUARK *ab initio* structure prediction pipeline, where the flowchart is depicted in Mortuza et al.^29^ C-QUARK uses a multiple sequence alignment generation tool, DeepMSA32, which is used for profile construction and contact-map prediction, and a deep learning-based and coevolution-based contact prediction module for residue contact-map prediction, combination, and selection. In the end, a contact potential term developed and carefully trained to balance its contribution with the other energy terms, guides the structure assembly simulations.

### Immunohistochemical analysis

Immunohistochemical investigations were performed on liver sections from the patient’s samples pre- and post-treatment with ataluren. Samples were fixed in formalin (10% [vol/vol]), then embedded in paraffin (FFPE), and cut into 3-μm sections. Two different proteins were investigated: Cytokeratin 19, as an experimental epithelial control marker, and LRBA, the target to test. Briefly, to verify tissue sections’ immunohistochemical reactivity and responsiveness, sample slides were incubated with antibody anti-cytokeratin 19 (clone A53-B/A2.26, Roche Ventana). Successively, other slides were incubated with the antibody anti-LRBA, produced in rabbit (Sigma-Aldrich, HPA023597). The antibody anti-LRBA diluted 1:200 in antibody diluent (Roche Ventana 760108) was incubated for 36 min at 36°C, using UltraBenchmark (Roche Ventana) instrument, according to the manufacturer’s standard protocol.

Moreover, as positive experimental control, sections from healthy samples of the histological archive were also incubated with antibodies, anti-cytokeratin 19 and anti-LRBA, respectively, in the same experimental conditions.

### Platelet measurement

To assess the evolution of the patient following ataluren treatment initiated on July 29, 2021, platelet evolution before and after the start of treatment was analyzed.

Data: Platelet values for this patient are available between January 5, 2021, and January 25, 2022 (date of transplantation).

Methods: The date of the first platelet measurement (January 5, 2021) was considered as day 0 (J0). The patient was considered to be under treatment starting from July 29, 2021. Linear regression models were used to analyze the effect of time on platelets, separately before and after the start of treatment. A multiple linear regression model was used over the entire period considered (from January 5, 2021, to January 25, 2022) to evaluate the effect of treatment and the effect of time on platelets.

Effect of time before treatment initiation: *p* = 0.96 -> not statistically significant • Effect of time after treatment initiation: *p* = 0.58 -> not statistically significant. The corresponding regression lines for both are represented on the graph.

(1) Over the entire period considered (from 5/01/2021 to 25/01/2022):
(2) Treatment effect (before/after): p < 0.001
(3) Time effect: p = 0.63

### Statistics

All data are given as means ± S.E.M. and was calculated by the one-way ANOVA test. A probability value of 0.05 was regarded as significant (GraphPad Software Prism version 6.0, Inc., La Jolla, CA, USA).

### Study approval

This study is a single patient protocol (Eudra CT 2021-003-004-41) approved by the Belgian Federal Agency for Medicines and Health Products (FAMHP) on 22 July 2021.

All human samples were collected and analyzed according to the standards of the ethical committee and the Declaration of Helsinki, following obtaining written consent. The patient gave written informed consent for skin biopsy, fibroblast culture, inclusion in the trial, and analysis on the explant liver. All protocols were approved by the Ethics Committee of the CHU de Liège approval CUP002 ATALUREN. Cells harboring the nonsense mutation c.5047 C>T 8p. (R1683X) in *LRBA* genes, were harvested from the patient biopsy.

## Data availability

Relevant information about data is available directly from the corresponding authors.

## Acknowledgments

The authors wish to thank Dr Sandra Giannini for reviewing and editing the manuscript in addition to useful discussions. The authors wish to express their gratitude to PTC Therapeutics Inc. for providing ataluren (Translarna) for the patient included in this study. The research leading to these results has received funding from the European Union - NextGenerationEU through the Italian Ministry of University and Research under PNRR - M4C2-I1.3 Project PE_00000019 "HEAL ITALIA" to I.P. and L.L. (University of Palermo), CUP B73C22001250006 and PRJ -0863 PRIN2022 to I.P. (University of Palermo) CUP B53D23008390006. The views and opinions expressed are those of the authors only and do not necessarily reflect those of the European Union or the European Commission. Neither the European Union nor the European Commission can be held responsible for them.

## Author contributions

Conceptualization: M.M. and I.P.; methodology: M.M., I.P., and L.L.; investigation: L.L., R.P., R.M., F.C., I.F., D.R., P.S.C., A.C., P.D., F.G., and M.T.; visualization: A.P., B.G., M.T., and P.S.C.; funding acquisition: I.P. and M.M.; project administration: I.P., L.L., and M.M.; supervision: I.P., L.L., and M.M.; writing – original draft: I.P., L.L., M.M., and M.T.; writing – review & editing: I.P., L.L., M.M., and P.S.C.

## Declaration of interests

The authors declare no competing interests.
